# The effect for Japanese workers of a self-help computerized cognitive behaviour therapy program with a supplement soft drink

**DOI:** 10.1186/s13030-017-0109-5

**Published:** 2017-09-19

**Authors:** Kentaro Shirotsuki, Yuji Nonaka, Keiichi Abe, So-ichiro Adachi, Shohei Adachi, Tomifusa Kuboki, Mutsuhiro Nakao

**Affiliations:** 1 Faculty of Human Sciences, Musashino University, 3-3-3, Ariake, Koto-ku, Tokyo, 135-8181 Japan; 2 Innovation Development Department, Suntory Global Innovation Center Limited, Osaka, Japan; 3 Medical corporation So-bun-kai, Clinic Adachi, Gifu, Japan; 4 Professor Emeritus, The University of Tokyo, Tokyo, Japan; 5 Department of Psychosomatic Medicine, Teikyo University Hospital, Tokyo, Japan

**Keywords:** Self-help, Computerized cognitive behaviour therapy, Workplace, L-carnosine

## Abstract

**Background:**

Computerized cognitive behaviour therapy (CCBT) programs can provide a useful self-help approach to the treatment of psychological problems. Previous studies have shown that CCBT has moderate effects on depression, insomnia, and anxiety. The present study investigated whether a supplement drink that includes L-carnosine enhances the effect of CCBT on psychological well-being.

**Methods:**

Eighty-seven participants were randomly allocated to a control group, CCBT, or CCBT with supplement drink. The CCBT and CCBT with supplement drink groups received six weekly self-help CCBT program instalments, which consisted of psycho-education about stress management and coping, behaviour activation, and cognitive restructuring. The CCBT group consumed a bottle of the supplement soft drink every morning through the 6 weeks. This program was delivered by an e-learning system on demand and also included a self-help guidebook. Seventy-two participants completed the program or were assess at the end of the study.

**Results:**

ANOVA revealed that there were significant interactions (times × groups) for POMS tension-anxiety and fatigue. The CCBT group showed significantly improved tension-anxiety scores, whereas the CCBT with drink group showed significant improvements on fatigue.

**Conclusion:**

The self-help CCBT program reduced the subjective experience of tension-anxiety in this group of workers. The addition of a supplement drink enhanced the effect of CCBT on fatigue, providing one possible approach to enhancement of such programs.

**Trial registration:**

This study was registered on September 2, 2016 at UMIN. The registration number is UMIN000023903.

## Background

Computerized cognitive behaviour therapy (CCBT) programs involve the effective delivery of evidence-based treatments over the Internet, using computers, tablets, or smartphones. CCBT is a self-help treatment. Self-help cognitive behaviour therapy (CBT) can provide a useful approach for the treatment of psychological problems. Previous studies show that self-help CBT programs have moderate effects on depressive symptoms and anxiety disorders [1, 2]. Additionally, self-help treatment such as CCBT can also address barriers to care such as limited availability of clinicians trained in evidence-based interventions [3]. Additionally, CCBT is effective for those patients who are reluctant to attend treatment in clinical settings due to stigma [4]. Self-help CBT programs have been used to address depression and anxiety [5–7] as well as insomnia [8]. Meta-analyses suggest that self-help CBT can be effective in the treatment of low to moderate severity of psychological difficulties [9, 10].

The WHO reports that the prevalence of mental disorders in the countries surveyed exceeds 10% [11]. Systematic reviews indicate that work-related psychological distress is related to mental disorders, including both depression and anxiety disorders [12, 13]. In addition, it is often argued that management of psychological distress is important for optimum work performance, regardless of clinical significance [14, 15]. Several recent studies examined the effects of web-based CBT programs among employees in Japan. Kimura et al. examined the effect of a brief CBT-based training program on work performance [16]. This combination of a group CBT session with web-based CBT homework significantly improved subjective work performance. Another study examined the effects of a brief CBT-based training program in terms of alleviating psychological distress among employees and facilitating self-evaluation of stress management skills. This program combined group CBT education with web-based CBT homework, and led to a moderate alleviation of symptoms for employees with clinically significant psychological distress [17]. These reports suggested that Self-help CBT does appear to be a moderately effective intervention for a range of common mental health difficulties in the workplace.

Additionally, previous studies have assessed the effect of eating supplement foodstuffs on health condition. Carnosine (β-alanyl-L-histidine) and related compounds, including homocarnosine and anserine together with N-acetylated forms, are common dipeptides found in mammals, birds, and fish [18–22]. Carnosine, which is present in meats such as chicken or beef, is readily absorbed intact into the jejunum, despite being a dipeptide [23]. It is metabolized by the enzyme carnosinase [24] and excreted via the kidneys [23]. Yamano et al. reported that daily intake of chicken extract that includes large amounts of imidazole dipeptides (carnosine and anserine) promotes recovery from mental fatigue, and these researchers concluded that the extract could be a candidate for use as an anti-fatigue food [25]. Smizu et al. showed that fatigue visual analogue scale (VAS) scores were significantly lower two to 8 weeks after administration of a drink containing imidazole dipeptides (400 mg) compared with placebo [26], making such a beverage another potential anti-fatigue food. Chengappa et al. examined the hypothesis that L-carnosine, an antioxidant and anti-glycation agent, would improve executive dysfunction, a cognitive domain associated with glutamate in patients with schizophrenia [27]. They suggested that L-carnosine merits further consideration as an adjunctive treatment to improve executive dysfunction in a patient population with psychiatric problems.

Based on these findings, low-intensity and easy-to-implement self-help CBT may yield improved mental health outcomes. It is possible that supplementation with L-carnosine may reinforce the effect of self-help CBT on work-place stress. The present preliminary investigation examined whether a supplement drink that included L-carnosine would enhance the effects of self-help CBT in the workplace. It was hypothesized that CCBT would improve anxiety, depression and fatigue and the use of supplement drink would enhances the effect of CCBT, especially on fatigue.

## Method

### Participants

Healthy volunteers were recruited from employees working at beverage, alcoholic beverage, and food manufacturing/sales companies in Tokyo, Japan. Participants included the employees of the group companies that created the supplement drink. Members directly belonging to the division related to this research did not participate in this study.

All participants were office workers and had full-time job work. Informed consent sessions were held in September 2014. The program took place from October to November of 2014.

Based on previous studies, it was assumed that the self-help CCBT has a moderate effect on psychological factors. From the results of power analysis, a power of 0.85 for medium effect for interaction was required. Therefore, we tried to collect 90 participants. In this research, research guidance and recruitment were done by e-mail, after which those who signed up for the study participated the informed consent session in which the purpose and procedures of the study were explained, and the 96 people who gave written concent were enrolled for study. Of these, six declined to participate and three were later excluded due to the exclusion criteria, which included systolic blood pressure less than 90 mmHg, pregnancy or possible pregnancy or lactation, participating in other studies, presence of internal diseases, history of cardiovascular disease, diabetes mellitus, or investigator-determined unsuitability. No individuals who had chronic diseases participate in this study. This left 87 employees available for the study. They were randomly allocated to a control group (*n* = 29), self-help CBT group (*n* = 29), or self-help CBT with supplement drink group (*n* = 29). Simple randomization was conducted by an independent study controller who had no direct contact with the participants. All personal information was completely managed by a company that was another secretariat for the protection of personal information. The company to be secretariat was originally independent of the participants and researchers.

**Fig. 1:**
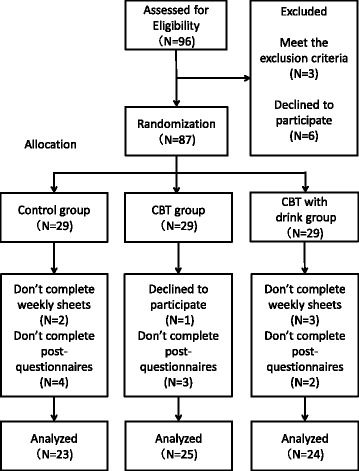
Participation flow chart. This flow chart shows the transition of participants. Ninety-six people signed up to participate. Before the start of the program an informed consent session was held, the purpose and procedures of the study were explained, and 96 participants provided their written informed consent. After the informed consent session, three people were excluded due to exclusion criteria and six people declined to participate. The remaining 87 employees were randomly allocated to a control group (*n* = 29), self-help CBT group (*n* = 29), or self-help CBT with supplement drink group (*n* = 29). Of the 87, 80 completed the relevant program and the pre- and post- sets of questionnaires. We also excluded two participants who did not complete weekly tasks and failed to submit weekly homework sheets. Additionally, six participants were excluded because they did not complete the post-questionnaires. At the end of the study, we analysed the data of 72 completers (control group: *n* = 23, CBT group: *n* = 25, CBT with supplement drink group: *n* = 24)

Of the 87 participants, 80 completed the relevant program and all of the pre- and post- sets of questionnaires. We also excluded two participants who did not complete the weekly tasks and failed to submit weekly homework sheets. Additionally, the days of six participants were excluded because they did not complete the post-questionnaires (Fig. 1). At the end of the study, we analysed the data of 72 completers (control group: *n* = 23, CBT group: *n* = 25, CBT with supplement drink group: *n* = 24). The completion rate was 82.75%. The age and gender composition of the groups was as follows: “control group; mean age = 38.35, SD = 8.83; 17 male and 6 female”, “CBT group; mean age = 35.44, SD = 10.29; 18 male, 7 female”, “CBT with supplement group; mean age = 37.88, SD = 9.15; 14 male, 10 female”.

### Protocol

All participants completed the questionnaires before and after the 6 week program. The CBT and CBT with drink group participants attended a course on mental health on two occasions (30 min each) before and after the program. Due to the nature of the intervention, participants were aware of their allocation status.

In preparation for the possible occurrence of an adverse event, a system was developed for giving treatment and doing follow-up until the event recovered or lightened. No adverse events were reported during or after the study.

### Control group

The control group recorded their mood state every day on a weekly monitoring sheet. They submitted their monitoring sheets to the study secretariat weekly.

### Self-help CBT program

The CBT and CBT with supplement drink groups received six weekly instalments of a self-help CBT program, consisting of psycho-education about stress management, stress coping, behaviour activation, and cognitive restructuring. This program was completely devoid of therapy contact or support and was delivered using an on demand e-learning system and a self-help guidebook titled “Stress ni makenai hon (in Japanese)” [28]. Participants watched weekly e-learning movie segments (5–10 min long) and read the corresponding sections of the guidebook. Weekly key words were provided during the movies and participants filled out the words (e. g., autumn) on a task sheet. They recorded their daily moods and weekly tasks on a monitoring sheet. Sheets were submitted weekly to the research secretariat. The contents of the CBT program are shown in Table 1.

**Table 1:** Contents of present self-help CBT program

No. 1	Understanding oneself
No. 2	Social support
No. 3	Coping with stress
No. 4	Behavioral activation: “To Do” lists
No. 5	Creating one’s own cognitive model
No. 6	Cognitive restructuring

**Table 2:** The data of pre-assessment of each value

	Control group (*N* = 23)		CBT group (*N* = 25)		CBT with drink group (*N* = 24)		
	Mean	SD	Mean	SD	Mean	SD	F-values
POMS-TA	10.09	5.23	14.84	8.76	13.83	7.23	2.81^†^
POMS-D	7.09	8.49	11.96	12.32	12.04	11.55	1.57
POMS-F	5.35a	4.09	9.84b	7.00	12.54c	7.01	7.99** a < b*, a < c**
SSAS	26.26	6.74	27.00	7.22	29.46	5.29	1.59
MSCL	1.78	2.04	1.92	2.27	3.17	2.35	2.79^†^
GSES	9.87	3.44	7.84	4.45	8.67	4.21	1.50

**p<.01, *p<.05, ^†^ *p*<.10

**Table 3:** The data of post-assessment of each value

	Control group (*N* = 23)		CBT group (*N* = 25)		CBT with drink group (*N* = 24)		
	Mean	SD	Mean	SD	Mean	SD	F-values
POMS-TA	11.83	6.55	12.36	6.13	12.58	5.52	0.10
POMS-D	7.83	9.12	11.52	9.65	9.83	10.64	0.85
POMS-F	7.70	5.50	8.96	6.91	9.29	5.34	0.47
SSAS	26.61	5.69	26.44	7.28	28.79	6.11	1.01
MSCL	1.55	1.97	2.04	2.23	2.79	2.65	1.71
GSES	10.35	3.65	8.04	4.49	9.33	4.23	1.87

### Supplement drink

The CBT with supplement drink group consumed one bottle of the supplement soft drink (100 ml) every morning throughout the 6 weeks. The drink contained 200 mg of L-carnosine. The safety of L-carnosine intake has been reported by Simizu et al. [26]. The study reported that intake of a drink containing imidazole dipeptides at both 200 mg/day (low dose) and 400 mg/day(high dose) for 8 weeks improved fatigue from daily activities. Additionally, there were no adverse events reported in the study. Other studies also verified the safety [29, 30]. Based on these reports of safety and effect, 200 mg of L-carnosine was used in this study. One bottle of the drink also contains 0.2 g protein, 18 g carbohydrate, and 40 mg sodium, but no fat. This drink did not include any other substance that might improve mood or fatigue. Consumption of the drink was recorded on the task sheet. The CBT self-help program was the same as that administered to the CBT group, as described above. The details of the content of the beverage were not explained before the start of the program. This drink was not a commercial item but was created for this research.

### Measures

#### Profile of Mood Scale (POMS) [31]

The Japanese version of POMS was administered before and after the program. In the present study, the Tension-Anxiety (TA), Depression (D), and Fatigue (F) subscales of the POMS were used. The Japanese version of the POMS has sound reliability and validity [31].

According to the original study, a score greater than or equal to 34 is used to delimit depression and 20 for mild depression for men, with corresponding scores of 28 and 18 for anxiety. Another study showed that the POMS has the reliability and validity necessary to measure the mood state of older adults [32]. Another study reported the average scores and standard deviation for the general population (POMS-TA: 12.0 ± 6.3; POMS-D: 11.4 ± 9.6; POMS-F: 9.3 ± 6.2, [33].

#### Somatosensory Amplification Scale (SSAS) [34]

The SSAS includes 10 items that ask the respondent to endorse a range of uncomfortable bodily sensations on an ordinal scale from 1 to 5. Nakao et al. reported high reliability and validity for the SSAS. The SSAS has item-to-scale correlations of 0.27 to 0.84 (*p* < 0.05), with adequate internal consistency (Cronbach alpha = 0.79) [34]. In addition, SSAS scores are positively correlated with the total number of somatic symptoms, degree of perceived psychosocial stress, and POMS tension-anxiety, depression, fatigue, and confusion scale scores. SSAS scores were higher in a psychosomatic group than in their comparison group [34]. The SSAS is a clinically useful tool for the evaluation of Japanese psychosomatic patients in terms of how patients experience and report symptoms as well as their functioning in various medical conditions.

#### Medical Symptom Checklist (MSCL) [35]

The MSCL assesses the frequency, degree of discomfort, and the degree of interference with daily activities. Following previous studies, we selected the following 16 common medical symptoms: Headache, eye symptoms, dizziness, diarrhea, nausea, constipation, abdominal pain, muscle soreness, shortness of breath, limb pain, arthralgia, insomnia, back pain, fatigue, palpitation, and tinnitus. The calculated total number of symptoms was used as an index of MSCL (maximum = 16, minimum = 0).

#### General Self Efficacy Scale (GSES) [36]

The GSES assesses the individual’s general self-efficacy across a variety of settings in everyday life. The GSES consists of 16 items, each scored either 0 (NO) or 1 (YES). The GSES has been shown to have good internal consistency and has reasonably high reliability and validity.

### Statistical analysis

First, we conducted ANOVA to reveal group differences for all measures. Second, we calculated effect size (Cohen’s *d*) to assess the degree of change in significantly reduced POMS factor scores. Third, we conducted ANCOVA (group × time) and ANOVA for the change values of the factors when there was a significant difference in baseline values for all measures.

## Results

The results of ANOVA showed that there was no significant difference in age among the three groups (*F*(2, 69) = 0.66, *p* = 51). Additionally, Kruskal-Wallis test also revealed that there was no significant difference in terms of sex ratio (*p* = .48).

ANOVA was used to examine group differences for all measures (Table 2 and 3). Comparison between baseline scores showed that there was a significant difference between groups for the POMS-F (*F*(2, 71) = 7.99, *p* < .01). On POMS-D, there was no significant main effect (*F*(2, 71) = 1.57, *p* = .21), but there was a tendency toward a main effect for the POMS-TA (*F*(2, 71) = 2.80, *p* = .06).

**Fig. 2:**
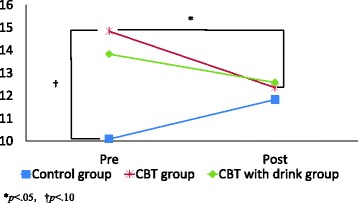
POMS-TA score change. POMS, Profile of mood states; TA, Tension-anxiety. The graph shows significant interaction between group and time of assessment for POMS TA (*F*(2, 69) = 3.04,*p* = .05). Post hoc tests using the Bonferroni method revealed that the CBT group significantly improved on POMS-TA (*p* < .05)

**Fig. 3:**
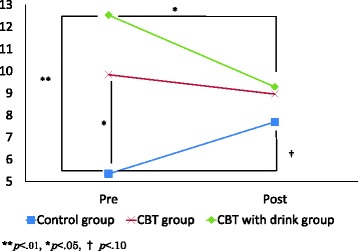
POMS-F scores change. POMS, Profile of mood states; F, Fatigue. There was significant interaction between group and time of assessment for POMS-F (*F*(2, 69) = 5.01,*p* < .01). Post hoc tests using the Bonferroni method revealed that the CBT with drink group significantly improved on POMS-F (*p* < .01)

There were also significant interactions between group and time of assessment for POMS-TA (*F*(2, 69) = 3.04, *p* = .05; Fig. 2) and POMS-F (*F*(2, 69) = 5.01, *p* < .01; Fig. 3). Post hoc tests using the Bonferroni method revealed that the CBT group significantly improved on the POMS-TA (*p* < .05) and that the CBT with drink group significantly improved on POMS-F (*p* < .01). We calculated effect sizes (Cohen’s *d*) to assess the degree of change in POMS-A scores for the CBT group and POMS-F scores for the CBT with drink group. Cohen (1988) proposed that effect sizes be categorized as follows: Small (0.20–0.49), medium (0.50–0.79), and large (0.80 or more) [37]. Using Cohen’s classification, the change in the POMS-TA score for the CBT group was a small effect (*d* = 0.37) and the change in the POMS-F score for the CBT with drink group was a medium effect (*d* = 0.60).

In the present study, there was a significant difference in pre-assessment POMS-TA and POMS-F. Therefore, we conducted ANCOVA (group × time) using the POMS-TA and POMS-F pre-treatment scores as co-variates. The results showed a significant interaction tendency for POMS-TA and POMS-F (*F*(3, 66) = 2.53, *p* = .06; *F*(3, 66) = 2.46, *p* = .07 respectively).

Additionally, examination of the change values of these factors by ANOVA revealed group differences in POMS-TA and POMS-F because there were significant differences at baseline for two of the psychological values, tension-anxiety and fatigue. There were significant main effects for the POMS TA and POMS F scores (*F*(2, 69) = 3.04, *p* = .05; *F*(2, 69) = 5.01, *p* = .01, respectively). Post hoc tests revealed that the CBT group had significantly improved the POMS-TA score (*p* < .05) whereas the CBT with drink group significantly improved the POMS-F score (*p* < .01).

There were no significant interactions for the other measures (i.e., POMS-D: *F*(2, 69) = .72, *p* = .48, SSAS: *F*(2, 69) = .36, *p* = .69, Medical Symptom Check List; *F*(2, 69) = .65, *p* = .52, and GSES: *F*(2, 69) = .41, *p* = .66).

## Discussion

The purpose of the present study was to examine the effects of a self-help CCBT program for healthy Japanese workers and to assess whether a supplement drink that contains L-carnosine would enhance the effect of self-help CBT. The present results indicated that the self-help CBT program improves feelings of tension and anxiety. Additionally, there is the possibility that the self-help CBT coupled with the supplement drink improved fatigue, which suggests that L-carnosine works to reinforce the effect of self-help CBT.

The effect size for the POMS-TA score of the CBT group was *d* = .32, and the effect size for the POMS-F score in the CBT with drink group was *d* = .52. One meta-analysis reported that the effect size of guided CCBT was *d* c = .38 [38]. Comparing these effect sizes, the present results are roughly consistent with prior results. Our self-help CBT consisted of e-learning and textbook study, without therapist support, focused on stress management, behavioral activation, and cognitive restructuring. The completion rate was 82.75%, a relatively high rate that suggests that the intervention was well-tolerated and user-friendly.

Following previous studies [24, 25], the present results indicate that foods and drinks containing L-carnosine may be effective for recovery from mental fatigue. For example, chicken extract contains large amounts of imidazole dipeptides (carnosine and anserine), which are natural antioxidants in meat. Chicken extract is used as a traditional remedy, with various aims [39]. In Asian countries, it is widely taken for attenuation of fatigue, recovery from stress, and increased mental efficiency [40].

It should be noted that there was no significant change in other psychological measures as a function of treatment (SSAS, Medical Check List, and GSES). This could be due to the fact that the present program did not specifically target psychosomatic responses or self-efficacy. The use of relaxation or deep breathing techniques might have a more robust effect on such targets. In addition, such a short program may have precluded much change in terms of participant perceptions of self-efficacy.

The CBT with drink group did not show significant improvement in their POMS-TA score. The baseline score for this subscale was relatively lower than that for the CBT with drink group, although this difference was not statistically significant. There was no difference between the groups in the POMS-TA post-score. This means that the baseline data difference may have influenced these results. Futures studies using blocked randomization may help clarify this possibility.

### Limitations

The present study has some limitations. First, there were significant differences at pre-assessment for some scales. The present study used a simple randomization design, which failed to result in equivalent group scores at pre-treatment. Future studies adopting block randomization may clarify the effects of the present self-help CBT program and L-carnosine. In addition, the present study did not set a group that took only a supplement soft drink. In future studies, it will be necessary to compare the effect of the supplement drink with the combined use of the supplement drink and CCBT. This improvement will clarify if the significant improvement in fatigue in our CBT with drink group was a psychological or biological affect. For example, comparison of a CBT with a supplement drink group with a CBT with placebo-drink group will be required.

Second, participants were relatively healthy employees. Future study should examine the effects of our program on patients with mental disorders. In terms of participants, the total sample size was 72, which is relatively low, and the power is .75. In future studies, it will be necessary to have a more adequate sample size, which would reinforce the present findings in terms of sample power and the significance of interaction. Also, no therapist guidance or support was provided. Potential augmentation effects of limited therapist support on our program should be examined. Furthermore, in the present study, all participants had a full-time job, however, we did not record the details of working hours and position. In future studies, assessment and consideration of these factors will be useful for clarifing the relation between the working environment and the effect of CCBT.

Third, participants in present study may have implicitly reported desirable results because they belonged to group companies of the one that created the supplement drink. It is difficult to completely eliminate bias by the participants. In future research, it will be important to consider this point.

## Conclusions

The present study investigated if a supplement drink that includes L-carnosine enhances the effect of CCBT on psychological well-being. The self-help CCBT program reduced the subjective experience of tension-anxiety by the workers. The addition of a supplement drink was shown to have enhanced the effect of CCBT on fatigue, providing one possible approach to the enhancement of such programs.

## Abbreviations

ANOVA: Analysis of variance
CBT: Cognitive behavioral therapy
CCBT: Computerized Cognitive behavioral therapy
D: Depression
F: Fatigue
GSES: General Self Efficacy Scale
MCS: Mental component summary
POMS: Profile of mood states
SD: Standard deviation
SSAS: Japanese version of Somatosensory Amplification Scale (SSAS)
V: Vigor
